# Morphology of the major salivary glands in ocelots (*Leopardus pardalis*): description of 02 cases

**DOI:** 10.29374/2527-2179.bjvm006126

**Published:** 2026-10-02

**Authors:** Letícia Victal Pereira, Anieli Vidal Stocco, Raquel Batista Junger de Carvalho, Carlos Augusto Santos Sousa, Paulo Souza, Marcelo Abidu-Figueiredo

**Affiliations:** 1 Departamento de Anatomia Animal e Humana, Universidade Federal Rural do Rio de Janeiro, Seropédica, RJ, Brazil.; 2 Programa de Pós-Graduação em Medicina Veterinária, Departamento de Medicina e Cirurgia Veterinária, Universidade Federal Rural do Rio de Janeiro, Seropédica, RJ, Brazil.; 3 Concremat Ambiental, Rio de Janeiro, RJ, Brazil.; 4 Instituto de Ciências Agrárias, Universidade Federal dos Vales do Jequitinhonha e Mucuri, Campus Unaí, Unaí, MG, Brazil.; 5 Departamento de Medicina Veterinária, Universidade Federal do Pampa, Campus Uruguaiana, Uruguaiana, RS, Brazil.

**Keywords:** anatomy, felines, salivary gland, anatomia, felinos, glândula salivar

## Abstract

The ocelot (*Leopardus pardalis*) is the largest species of its genus and, despite its wide distribution throughout the Americas, it is included on the main list of endangered species. Its conservation has been widely studied, but there is a lack of studies regarding its morphology. The morphology, measurements, and location of the salivary glands in wild felids are fundamental, as they provide basic information for understanding the biology of these species. This study was approved by the Research Ethics Committee of the Federal Rural University of Rio de Janeiro (018/2017). Anatomical dissections were performed on the cadavers of two adult female ocelots. In the specimens studied, the parotid (PG), mandibular (MG), molar (MolG), sublingual (SLG), and zygomatic (ZG) salivary glands were identified in both antimeres. The length, width, thickness, and weight of the glands were measured, revealing individual variations among the analyzed specimens. Information on the anatomy of salivary glands in wild animals is scarce. The description of these glands in wild felids is important, as it may facilitate clinical treatments and provide information for professionals working in zoos and conservation units.

## Resumo

A jaguatirica (*Leopardus pardalis*) é a maior espécie deste gênero e apesar de ter ampla distribuição nas Américas, está incluída na lista principal de espécies ameaçadas de extinção. Sua conservação é amplamente estudada, mas há uma carência de estudos sobre sua morfologia. A morfologia das glândulas salivares, suas medidas e localização em felinos selvagens são fundamentais, pois fornecem suporte básico para o conhecimento da biologia dessas espécies. Este estudo foi aprovado pelo Comitê de Ética em Pesquisa da Universidade Federal Rural do Rio de Janeiro (018/2017). Dissecções anatômicas foram realizadas em cadáveres de duas fêmeas adultas de jaguatirica. Nos espécimes estudados, as glândulas salivares parótida (GP), mandibular (GM), molar (GMol), sublingual (GSL) e zigomática (GZ) foram identificadas em ambos os antímeros. Foram realizadas mensurações de comprimento, largura, espessura e peso das glândulas, evidenciando variações individuais entre os espécimes analisados. São escassas as informações sobre a anatomia das glândulas salivares em animais silvestres. A descrição dessas glândulas em felinos silvestres é importante, pois podem facilitar tratamentos clínicos, gerando informações para profissionais que atuam em zoológicos e unidades de conservação.

## Introduction

Comparative anatomy is the science that studies the morphology and anatomical structures of living organisms, establishing comparative and evolutionary analyses among them (Kardong, 2016). The ocelot, *Leopardus pardalis* (Carnivora: Felidae), is a medium-sized mammal, weighing 8-15 kg, with long legs and a slender body, measuring from 50 cm to 1 m in length (Oliveira & Cassaro, 2005).

The ocelot is currently is included in the Red Book of Brazilian Fauna Threatened by vulnerable, by the Intituto Chico Mendes de Conservação da Biodiversidade (ICMBio), and listed as been assessed for The IUCN Red List of Threatened Species in 2014, as least concern (Caso et al., 2015). However, wild felids felines such as *Leopardus pardalis* can be considered important for conservation of the ecosystems they live in due to their abundance and ecological significance (Machado et al., 2017).

The anatomy of domestic felines has been neglected in the veterinary anatomical literatures, as most studies have focused on the dog as a Carnivora representative. Information on wild felids is even scarcer and often remains non-existent. Salivary glands secrete saliva, which plays an important role in food mastication (El-Kordy et al., 2014; Mohammadpour, 2010).

The salivary glands are classified as major and minor. In most animals, there are three pairs of major salivary glands: mandibular, sublingual, and parotid glands. In the felids, two other glands, the molar and a zygomatic gland, are considered part of the salivary system. The major salivary glands are large and located some distance from the oral cavity, and their excretory ducts are long. The minor salivary glands are located in the walls of the oral cavity and oropharynx and have very short ducts. They are named based on location i.e., labial, lingual, oral and palatine (Dyce et al., 2019).

Information on the morphology, measurements, and location of the salivary glands, in wild species is fundamental, as it provide basic information for understanding the biology of these species. These data can contribute to conservation strategies, clinical and surgical treatments and preservation of endangered species; changes in these glands such as sialocele and neoplasms can cause dysphagia and anorexia.

The objective of this study was to describe the anatomy and measurements of the major salivary glands of the ocelot.

## Material and methods

This study was approved by the Research Ethics Committee of the Federal Rural University of Rio de Janeiro (018/2017). Anatomical dissections were performed on the cadavers of two adult female ocelots. The specimens were obtained from the collection of the Laboratory of Teaching and Research in Morphology of Domestic and Wild Animals, collection of the Department of Animal and Human Anatomy, Federal Rural University of Rio de Janeiro. For fixation of the specimens, the thorax was first opened and dissected, and a No. 5 urethral catheter was introduced into the thoracic aorta. The arterial system was then "flushed" with 0.9% NaCl solution, and the cadaver was fixed with 10% formalin solution. Next, an aqueous solution(1:1dilution) of Petrolátex S-65 (RefineryDuque de Caxias-REDUC-Petrobras, Duque de Caxias, RJ) was injected together with a dye (Xadrez®). Finally, the specimens were immersed in a 500-L low density polyethylene box containing 10% formaldehyde solution to complete the latex polymerization process. At least seven days after latex injection, the cadavers were washed under running water. The salivary glands were dissected following the anatomical planes, preserving their syntopic relationships with the other structures. The glands were photographed, removed, measured and weighed. The nomenclature was based on the International Committee on Veterinary Gross Anatomical Nomenclature (World Association of Veterinary Anatomists, 2017).

## Results

### Gross anatomy

In the specimens studied, we identified the parotid, mandibular, molar, sublingual, and zygomatic salivary glands in both antimeres. The parotid salivary gland was located in the retromandibular fossa, caudal to the ramus of the mandible and ventral to the base of the ear. It was triangular (semilunar) in shape, with its base lying dorsally and its apex situated ventrally. The base was concave and enclosed the base of the external ear. The gland was loosely lobulated and had a notch dorsally. It possessed three borders: rostral, caudal, and dorsal. The parotid duct originated from the convergence of several small ducts and passed rostrally within the lateral facial fascia over the masseter muscle (Figure 1A).

**Figure 1:**
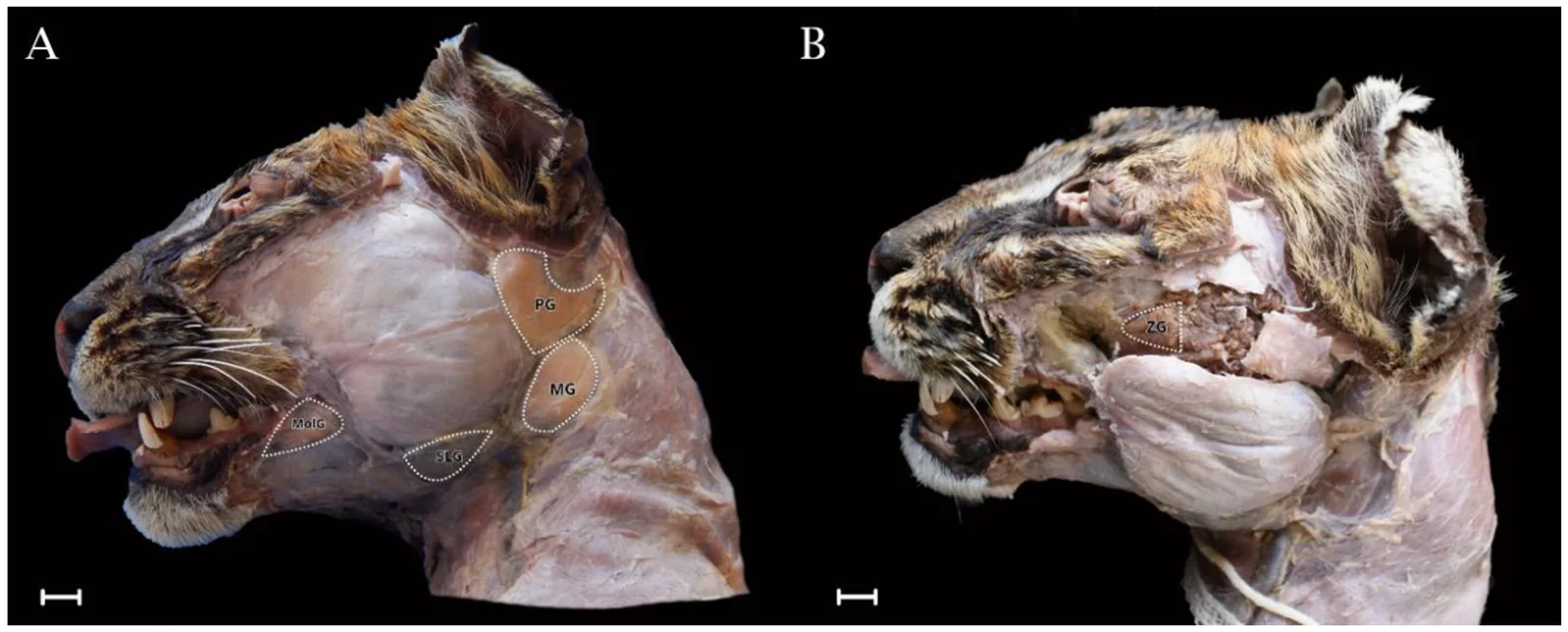
(A) Digital photomacrograph of the left lateral aspect of a female ocelot (*Leopardus pardalis*). Parotid gland (PG), mandibular gland (MG), sublingual gland (SLG), and molar gland (MolG). Scale bar: 1 cm; (B) Digital photomacrograph of the left lateral aspect of a female ocelot (*Leopardus pardalis*). Zygomatic gland (ZG). Scale bar: 1 cm.

The mandibular salivary gland was ovoid, with its wider extremity directed dorsally. It occupied a superficial position caudal to the ramus of the mandible and ventral to the parotid salivary gland. It lay caudal to the masseter muscle and the mandibular lymph node. Its cranioventral pole was related to the major portion of the sublingual salivary gland (Figure 1).

The sublingual salivary gland was elongated and triangular in outline, with its base directed caudally. It had a large caudal portion and a tapering rostral portion. The large caudal portion of the gland ay on the lateral aspect of the caudal part of the digastric muscle. It then crossed the dorsal border of this muscle, where the tapering rostral portion of the gland was situated between the digastric muscle and the ventral border of the masseter muscle, medial to the mandible (Figure 1A).

The zygomatic salivary gland was the only deeply located major salivary gland in the ocelot. It was pyramidal, compact, and glandular. Laterally, it was covered by the zygomatic arch (Figure 1B).

The molar gland was elongated in shape and was located between the orbicularis oris muscle and the mucous membrane of the lower angle of the mouth, near the oral commissure. The dorsal border of the molar gland was attached to the masseter muscle. The ventral border was located close to the buccal part of the buccinator muscle. The molar gland had a brownish-gray, granular appearance (Figure 1A).

### Measures

The results of the measurements obtained in both animals are represented in Table 1.

**Table 1:** Morphometric measurements and weights of the salivary glands of two ocelots (*Leopardus pardalis*).

**Salivary glands**	**Measures**	**Animal 01**		**Animal 02**	
		**left**	**right**	**left**	**right**
**Parotid gland**	Length	3.88 cm	3.73 cm	4.43 cm	4.28 cm
	Width	2.19 cm	1.92 cm	1.82 cm	1.54 cm
	Thickness	0.45 cm	0.46 cm	0.47 cm	0.49 cm
	Weight	2.20 g	2.20 g	1.90 g	1.90 g
**Zygomatic gland**	Length	2.86 cm	2.50 cm	1.79 cm	1.49 cm
	Width	1.11 cm	1.44 cm	1.48 cm	1.59 cm
	Thickness	0.53 cm	0.67 cm	0.68 cm	0.76 cm
	Weight	0.90 g	1.10 g	0.80 g	0.80 g
**Mandibular gland**	Length	2.77 cm	2.82 cm	3.27 cm	3.33 cm
	Width	1.99 cm	1.95 cm	1.95 cm	1.99 cm
	Thickness	0.90 cm	0.66 cm	0.85 cm	0.69 cm
	Weight	2.60 g	2.60 g	2.80 g	2.80 g
**Sublingual gland**	Length	1.39 cm	1.40 cm	1.31 cm	1.33 cm
	Width	2.11 cm	2.14 cm	1.90 cm	1.94 cm
	Thickness	0.35 cm	0.38 cm	0.10 cm	0.23 cm
	Weight	0.90 g	0.90 g	0.30 g	0.30 g
**Molar gland**	Length	2.25 cm	2.34 cm	1.87 cm	1.97 cm
	Width	0.79 cm	0.57 cm	0.79 cm	0.57 cm
	Thickness	0.33 cm	0.33 cm	0.29 cm	0.30 cm
	Weight	0.50 g	0.40 g	0.40 g	0.40 g

Legend: (cm) centimeter, (g) grams.

## Discussion

The anatomical description of the salivary glands of wild species is fundamental, as it provides basic knowledge of the biology of these specie. This information can contribute to conservation strategies, clinical treatments, and the preservation of endangered species.

Abouelela et al. (2022) studied the morphology and measurements of the major salivary glands of domestic cats. The authors observed that the parotid gland appeared as a triangular, flat, lobulated gland, with a length 3.53 ± 0.08 cm, and a width 3.2 ± 0.05 cm. The mandibular salivary gland appeared as a symmetrically spherical, thick, smooth, compact gland on both sides, directly beneath the subcutaneous fascia, with a length 2.5 ± 0.05 cm, and a width 1.4 ± 0.05 cm. On the other hand, the sublingual gland was elliptical thin gland with length 1.23 ± 0.08 cm, and width 3.1 ± 0.08 cm. The zygomatic salivary gland was the only deep one in the feline salivary glands. It was pyramidal, compact, and glandular with its apex directed ventrally and its base directed dorsally. Its length was 1 ± 0.11 cm, and width 1.96 ± 0.08 cm. The molar salivary gland was quadrilateral elongated granular in shape with length 0.36 ± 0.08 cm, and width 2.03 ± 0.08 cm.

Costa et al. (2021) described the shape and measurements of the molar gland in a lioness. The length, width, and depth of the glands were obtained. The molar gland had an elongated rectangular. The right molar gland measured 4.6 × 0.9 × 0.45 cm, with the width measured at its center. The cranial portion was 0.8 cm wide, and the caudal portion was 1.3 cm wide. The left molar gland measured 5.3 × 1.1 × 0.4 cm, with the cranial portion measuring 0.7 cm in width and the caudal portion measuring 1.3 cm in width.

Mohammadpour (2009) described the shape and size of the zygomatic and molar salivary glands in domestic shorthair cats. The zygomatic gland was with an oval shape with indented borders. The mean length of the gland was 0.81 ± 0.14 cm and 0.84 ± 0.09 cm on the left and right sides, respectively in the rostrocaudal direction. The mean width of the gland was 1.94 ± 0.11 cm (left side) and 1.91 ± 0.08 cm (right side) in dorsoventral direction. The molar gland was elongated. The mean length of the molar gland was 2.04 ± 0.14 cm and 1.99 ± 0.11 cm and the mean width was 0.59 ± 0.07 cm and 0.58 ± 0.08 cm on the left and right sides, respectively.

Santos et al. (2010) described the morphology of the mandibular salivary glands of coatis (*Nasua nasua*) and observed that these glands were located caudoventral to the masseter muscle and were partially covered by the parotid glands. They had a mean length of 2.9 cm, a width of 1.5 cm, and a thickness of 0.8 cm.

Santos et al. (2012) described the morphology of the parotid gland in two species of procyonids: the crab-eating raccoon (*Procyon cancrivorus*) and the coati (*Nasua nasua*) and observed that the parotid gland in the coati had a U shape, while that of the crab-eating raccoon had a Y shape, due to its two dorsal extremities that followed the shape of the auricular cartilage. These findings differ from those observed in ocelots, whose parotid glands had an inverted pyramid shape with a central indentation.

Pereira et al. (2012) characterized the morphology of the major salivary glands of crab-eating raccoon (*Procyon cancrivorus*) and observed that the major salivary glands of this species comprise the parotid, mandibular, sublingual and zygomatic glands. These findings are partly similar to those obtained in the present investigation, although in the ocelot, in addition to these glands, the molar salivary gland was also observed.

Pereira and Faria Júnior (2018) described the morphology of the major salivary glands in the crab-eating-fox (*Cerdocyon thous*) and observed that the salivary glands (parotid, mandibular and sublingual) in this species are distributed in the facial region and formed by a series of rectilinear ducts that converge toward the terminal secretory portions. These results are partly similar to those obtained in the present investigation, however in the ocelot, in addition to these glands, the molar and zygomatic salivary glands were observed.

In the present report, the results revealed that the major salivary glands of the ocelot included the parotid, mandibular, sublingual, molar and zygomatic glands, similar to those observed in the domestic cat (Abouelela et al., 2022) and partially similar to those observed in *Puma concolor* (Pereira et al., 2020), in which the authors did not report the zygomatic gland.

Salivary glands are accessory organs of the digestive system that play an important role in the food digestion. In addition, their secretions help lubricate food, facilitating chewing and swallowing (Amano et al., 2012). However, there is little information on the anatomical aspects of these organs in Neotropical wild felids.

Salivary gland diseases are well characterized in human medicine but are uncommonly reported in dogs. Lieske and Rissi (2020) described the clinical and pathologic features of 179 canine salivary gland biopsies. The main reason for sample submission was regional swelling (107 cases; 59.7%). Extraoral (major) salivary glands were affected in 125 cases (69.8%), and oral (minor) salivary glands were affected in 43 cases (24%).

Salivary glands can be affected by tumors (Barrantes Murillo et al., 2025), glandular duct stenosis (Vallefuoco et al., 2011), and sialocele (Bassanino et al., 2019). Therefore, information about the anatomy of these glands may support surgical and clinical treatments.

## Conclusion

The report described the salivary glands (parotid, mandibular, molar, sublingual, and zygomatic) in two adult female Leopardus pardalis specimens. There were differences in the morphology of the glands. The results reinforce the need for further studies on the species due to the scarcity of available information on Neotropical wild felids. In addition, the data obtained may contribute to future research and to improvements in clinical, surgical, and conservation management, assisting professionals involved in the health and preservation of this species.
